# Racial and Ethnic Differences in Health Care Experiences for Veterans Receiving VA Community Care from 2016 to 2021

**DOI:** 10.1007/s11606-024-08818-3

**Published:** 2024-05-31

**Authors:** Sudarshan Krishnamurthy, Yaming Li, Florentina Sileanu, Utibe R. Essien, Megan E. Vanneman, Maria Mor, Michael J. Fine, Carolyn T. Thorpe, Thomas Radomski, Katie Suda, Walid F. Gellad, Eric T. Roberts

**Affiliations:** 1 Department of Internal Medicine, Wake Forest University School of Medicine, Winston-Salem, NC USA; 2 VA Center for Health Equity Research and Promotion, VA Pittsburgh Healthcare System, Pittsburgh, PA USA; 3 VA Center for the Study of Healthcare Innovation, Implementation, and Policy, VA Greater Los Angeles Healthcare System, West Los Angeles, CA USA; 4 David Geffen School of Medicine, University of California Los Angeles, Los Angeles, CA USA; 5 Decision Enhancement and Analytic Sciences Center, VA Informatics, VA Salt Lake City Health Care System, Salt Lake City, UT USA; 6 Department of Internal Medicine, University of Utah School of Medicine, Salt Lake City, UT USA; 7 Department of Population Health Sciences, University of Utah School of Medicine, Salt Lake City, UT USA; 8 Division of General Internal Medicine, University of Pittsburgh School of Medicine, Pittsburgh, PA USA; 9 Eshelman School of Pharmacy, University of North Carolina, Chapel Hill, NC USA; 10 Division of General Internal Medicine, Perelman School of Medicine at the University of Pennsylvania, Philadelphia, PA USA

**Keywords:** Veteran Affairs, community care, disparities

## Abstract

**Background:**

Prior research documented racial and ethnic disparities in health care experiences within the Veterans Health Administration (VA). Little is known about such differences in VA-funded community care programs, through which a growing number of Veterans receive health care. Community care is available to Veterans when care is not available through the VA, nearby, or in a timely manner.

**Objective:**

To examine differences in Veterans’ experiences with VA-funded community care by race and ethnicity and assess changes in these experiences from 2016 to 2021.

**Design:**

Observational analyses of Veterans’ ratings of community care experiences by self-reported race and ethnicity. We used linear and logistic regressions to estimate racial and ethnic differences in community care experiences, sequentially adjusting for demographic, health, insurance, and socioeconomic factors.

**Participants:**

Respondents to the 2016–2021 VA Survey of Healthcare Experiences of Patients-Community Care Survey.

**Measures:**

Care ratings in nine domains.

**Key Results:**

The sample of 231,869 respondents included 24,306 Black Veterans (mean [SD] age 56.5 [12.9] years, 77.5% male) and 16,490 Hispanic Veterans (mean [SD] age 54.6 [15.9] years, 85.3% male). In adjusted analyses pooled across study years, Black and Hispanic Veterans reported significantly lower ratings than their White and non-Hispanic counterparts in five of nine domains (overall rating of community providers, scheduling a recent appointment, provider communication, non-appointment access, and billing), with adjusted differences ranging from − 0.04 to − 0.13 standard deviations (SDs) of domain scores. Black and Hispanic Veterans reported higher ratings with eligibility determination and scheduling initial appointments than their White and non-Hispanic counterparts, and Black Veterans reported higher ratings of care coordination, with adjusted differences of 0.05 to 0.21 SDs. Care ratings improved from 2016 to 2021, but differences between racial and ethnic groups persisted.

**Conclusions:**

This study identified small but persistent racial and ethnic differences in Veterans’ experiences with VA-funded community care, with Black and Hispanic Veterans reporting lower ratings in five domains and, respectively, higher ratings in three and two domains. Interventions to improve Black and Hispanic Veterans’ patient experience could advance equity in VA community care.

**Supplementary Information:**

The online version contains supplementary material available at 10.1007/s11606-024-08818-3.

## INTRODUCTION

There are known racial and ethnic health care disparities among Veterans who receive care within the Veterans Health Administration (VA).^1–5^ These disparities extend to patient-reported experiences with care, which capture patient-centered measures of health care quality.^6^ Among VA healthcare system enrollees, research found that Black Veterans reported poorer experiences with care, including communication with physicians, compared to non-Hispanic White Veterans.^7^ Additionally, research identified disparities in Veterans’ experiences with care in VA facilities that disproportionately serve Veterans from underrepresented groups,^8^ with Veterans being more likely to report negative experiences with care at VA facilities serving higher proportions of Black and Hispanic Veterans.^9^

Although numerous studies examined health care disparities within the VA, less is known about the experiences of Veterans receiving care outside of the VA healthcare system. A growing number of Veterans now receive VA-funded care from community providers (i.e., outside of the VA) because of two major policy reforms: the Veterans Access, Choice, and Accountability Act of 2014 (Choice Act) and the VA Maintaining Internal Systems and Strengthening Integrated Outside Networks Act of 2018 (MISSION Act). The Choice Act expanded Veterans’ ability to receive VA-funded care from community providers if they could not obtain timely care within the VA healthcare system or lived far from or experienced hardship getting to a VA healthcare facility. The MISSION Act broadened eligibility for and made these community care programs permanent.^10^ Over 2.6 million Veterans—nearly one in three Veterans enrolled in the VA healthcare system—were referred to community providers in the 18 months after the passage of the MISSION Act.^10,11^ Veterans are eligible to use community care for a range of primary and specialty care services (e.g., physical therapy, orthopedic care, and ophthalmology), and often use VA-funded community care in addition to care within the VA healthcare system.^11,12^

Community care programs were intended to improve Veterans’ access to timely and high-quality health care.^13,14^ However, concerns remain about whether Veterans from minoritized racial and ethnic groups have equitable experiences navigating community care and accessing high-quality community providers.^15,16^ These concerns are especially salient given the pervasive and entrenched factors that give rise to and perpetuate racial and ethnic disparities in health care access, quality, and outcomes nationally.^3,17^ Although a few studies have examined Veterans’ experiences with community care,^18,19^ we are not aware of research that quantifies racial and ethnic differences in Veteran-reported experiences with VA community care.

The objective of this study was to use data from the VA’s Survey of Healthcare Experiences of Patients – Community Care Survey (SHEP-CCS) to examine Veterans’ experiences with community care by race and ethnicity between 2016 and 2021.

## METHODS

### Study Design and Data Sources

We conducted an observational analysis of Veterans’ ratings of health care experiences in community care settings by race and ethnicity using the VA SHEP-CCS for the period 2016–2021.^20^ The SHEP-CCS is a mixed-mode (internet/mail) survey administered to Veterans who received VA-funded community care over the prior 3 months. The survey asks Veterans to rate their experiences across nine domains.^19,21,22^ Sampling for the SHEP-CCS is random within strata, which reflect the type of care received (e.g., primary care, psychiatric care, other subspecialty care). We linked respondent-level data from the SHEP-CCS to data from the Centers for Medicare and Medicaid Services (CMS) to identify Medicare and Medicaid enrollment, the VA Corporate Data Warehouse (CDW) to obtain information on demographics, VA priority group status, health conditions, geography, and the Veterans Integrated Services Network (VISN) where care was received. VISNs are regional divisions of the Veterans Health Administration that manage VA medical centers and other medical facilities (nationally, there are 18 VISNs).^23^ The VA Pittsburgh Healthcare System Institutional Review Board approved this study.

### Study Sample

SHEP-CCS had a response rate of 30.7% during the study period, in line with other surveys of patient-reported care experiences.^24^ A total of 233,634 respondents to the SHEP-CCS were identified from 2016 to 2021. We excluded 188 respondents who could not be linked to VA CDW data; 1093 respondents who resided outside of the 50 US states or Washington, D.C.; and 484 respondents without county-level geographic identifiers (needed to measure county-level covariates). From this sample, we analyzed differences in community care experiences based on ethnicity (*n* = 16,490 Hispanic and 200,725 non-Hispanic Veterans) and race (*n* = 24,306 Black/African American and 180,313 White Veterans).

### Outcomes

We examined experiences in nine domains: overall satisfaction with community care, overall rating of the provider, eligibility determination for VA community care, first appointment access, scheduling a recent appointment, provider communication, care coordination, non-appointment access (e.g., after-hours access to providers, waiting time in the office), and billing.^21^ We followed domain-item groupings for the VA-SHEP survey to combine responses to individual survey items into domain scores (see Appendix for details). Respondent-level scores were constructed as equally weighted means of ratings of domain items. Items were linearly converted to 0–100-point scales before aggregation into scores. Higher scores represent greater satisfaction with care.

### Independent Variables

We analyzed SHEP respondents according to their self-reported race and ethnicity. Race and ethnicity are social constructs and reflect the influence of social, political, and economic forces that lead to institutional inequity and interpersonal discrimination.^25–27^ We compared community care experiences of Veterans by race (comparing those who identified as Black or African American vs. White, regardless of ethnicity) and ethnicity (comparing those who identified as Hispanic vs. non-Hispanic, regardless of their race). Veterans who identified as Black/African American in addition to other racial groups were analyzed as Black. We did not separately analyze care experiences among racial or ethnic groups with smaller representation in the SHEP, such as American Indian or Alaska Native and Asian Veterans, because smaller sample sizes limited our ability to make meaningful comparisons.

### Covariates

We assessed age, gender, health, insurance and socioeconomic status, rural residence, county-level supply of health care providers, and type of community care received. To measure health status, we used the Elixhauser Comorbidity Index^28^ along with separate indicators for the presence of a serious mental illness (i.e., bipolar disorder, major depression, post-traumatic stress disorder, schizophrenia, or psychosis) and substance use disorder (i.e., substance use disorder related to drug or alcohol use). We identified these conditions using diagnoses on Veterans’ health care records in the VA CDW (care provided within VA) and in VA Program Integrity Tool files (administrative claims for community care) in the 2 years preceding the SHEP-CCS survey. We measured socioeconomic status and insurance using VA priority group status (which reflects Veterans’ income and service-connected disabilities^29^) and with indicators for enrollment in Medicaid, Medicare, Medicare Part D, and Medicare Advantage.^30,31^ Insurance is correlated with socioeconomic status and may impact access to care outside of the VA healthcare system. We included county-level measures of urban vs. rural residence (large metropolitan area, small metropolitan area, micropolitan area, and rural) and supply of community physicians per 1000 county residents.^32^ Finally, we included indicators for category of community care received: primary care, subspecialty care, surgical care, eye care, acupuncture, psychiatric care, and other care.

### Statistical Analyses

We plotted unadjusted ratings of community care experiences to examine trends and racial and ethnic differences in ratings over the study period. Next, for each domain score, we ran three sets of respondent-level linear regression models to estimate racial and ethnic differences in community care experiences. We constructed sequential models, guided by the National Academy of Medicine’s framework for examining health care disparities. According to this framework, disparities represent racial or ethnic differences that are not explained by group differences in health status or care needs. The framework considers how geographic, socioeconomic, and insurance factors may mediate racial and ethnic disparities in care.^33^

Accordingly, Model 1 adjusted for demographic factors (age and sex), health status, indicators for the category of community care received, and year fixed-effects. Model 2 adjusted for all variables in the first model as well as rurality and county-level supply of physicians. Model 3 further adjusted for education, along with socioeconomic and insurance factors (included together because many differences in insurance coverage are income-related). Sequential adjustment for covariates allowed us to quantify the extent to which differences in ratings persisted after adjustment for geography, socioeconomic status, and insurance. We also conducted a sensitivity analysis that controlled for individual Elixhauser comorbidities instead of a linear comorbidity index.

We conducted a secondary analysis to explore whether Veterans differed in their likelihood of reporting high vs. low ratings of care by race or ethnicity.^9,34^ For each domain score, we estimated logistic regression models to test for differences by race or ethnicity in the probability of rating care on that domain at or above 90th percentile (high rating) or at or below 10th percentile (low rating). Percentiles were constructed for each patient experience domain among all SHEP-CCS respondents. We estimated marginal differences in the probability that Black vs. White or Hispanic vs. non-Hispanic Veterans reported high vs. low ratings of care, adjusting for all covariates in Model 3.

All estimates were weighted to account for survey sampling and non-response using STATA version 15. Statistical tests were conducted using a two-tailed 5% type-I error rate.

## RESULTS

### Sample Characteristics

Our sample comprised 16,490 (7.6%) Hispanic and 200,725 (92.4%) non-Hispanic Veteran-year observations, which when weighted represented 1,419,630 and 13,851,118 Veteran-years in the population of community care users, and 24,306 (11.9%) Black/African American and 180,313 (88.1%) White Veteran-year observations, which when weighted represented 2,027,820 and 12,183,563 Veteran-years in the population.

In the survey-weighted sample, the mean (SD) age was 54.6 (15.9) years among Hispanic Veterans and 61.2 (15.1) years among non-Hispanic Veterans (Table 1). The mean (SD) age was 56.5 (12.9) years among Black Veterans and 62.0 (15.4) years among White Veterans. The distribution of Elixhauser comorbidities was similar across racial and ethnic groups, although higher proportions of Hispanic and Black Veterans were diagnosed with a serious mental illness (40.0% among Hispanic Veterans and 38.8% among Black Veterans vs. 30.1% among non-Hispanic and 29.0% among White Veterans). Higher proportions of Black and Hispanic Veterans were enrolled in Medicaid, and smaller proportions had Medicare. Black and Hispanic Veterans were slightly more likely to use community care for acupuncture and psychiatric care but less likely to use community care for eye care and other medical subspecialty care compared with White and non-Hispanic Veterans, respectively.

**Table 1:** Characteristics of Veterans in the Survey of Healthcare Experience of Patients (SHEP) Survey Administered to Community Care Recipients, 2016–2021

	Hispanic ethnicity ^b^		Race ^b^	
Characteristic ^a^	Hispanic ethnicity *n* = 16,490 Weighted sample = 1,419,630 ^c^	Not Hispanic ethnicity *n* = 200,725 Weighted sample = 13,851,118 ^c^	Black or African American *n* = 24,306 Weighted sample = 2,027,820 ^c^	White *n* = 180,313 Weighted sample = 12,183,563 ^c^
Demographics				
Age, mean [SD] in years ^d^	54.6 [15.9]	61.2 [15.1]	56.5 [12.9]	62.0 [15.4]
Gender ^d^				
Female	1396 (14.7%)	17,005 (13.2%)	4029 (22.5%)	12,720 (11.4%)
Male	15,094 (85.3%)	183,720 (86.8%)	20,277 (77.5%)	167,593 (88.6%)
Health status				
Elixhauser comorbidities ^e^				
< 3 conditions	10,054 (69.2%)	123,545 (66.9%)	13,866 (64.4%)	111,154 (66.9%)
3 to 4 conditions	4163 (20.9%)	49,696 (21.8%)	6740 (24.0%)	44,346 (21.7%)
> 5 conditions	2273 (9.9%)	27,484 (11.3%)	3700 (11.6%)	24,813 (11.4%)
Substance use disorder ^f^	1201 (8.0%)	12,608 (6.7%)	2309 (8.6%)	10,521 (6.4%)
Serious mental illness ^g^	5835 (40.0%)	52,634 (30.1%)	8594 (38.3%)	45,238 (29.0%)
Education, insurance, and socioeconomic status (SES)				
Education				
Less than high school	1067 (3.3%)	10,567 (3.6%)	991 (2.6%)	10,752 (4.1%)
High school	4217 (19.7%)	57,580 (24.5%)	6106 (20.5%)	53,744 (25.7%)
College or more	11,131 (76.6%)	131,925 (71.6%)	17,100 (76.5%)	115,165 (69.9%)
Missing	75 (0.4%)	653 (0.3%)	109 (0.4%)	652 (0.3%)
VA priority group status ^h^				
Groups 1–4	12,141 (79.4%)	125,203 (69.2%)	17,788 (78.3%)	109,733 (67.6%)
Group 5	2688 (12.1%)	41,533 (16.7%)	4,300 (13.7%)	38,306 (17.3%)
Groups 6–8 or missing	1661 (8.5%)	33,989 (14.1%)	2218 (8.0%)	32,274 (15.1%)
Enrolled in Medicaid ^i^	1236 (8.1%)	12,437 (6.8%)	2238 (9.5%)	10,452 (6.3%)
Enrolled in Medicare ^i^	11,572 (46.1%)	160,232 (61.4%)	15,871 (47.0%)	147,838 (63.8%)
Enrolled in a Medicare Part D plan ^j^	3729 (14.1%)	46,107 (17.4%)	4105 (12.0%)	43,949 (18.8%)
Enrolled in a Medicare Advantage Plan	3103 (11.5%)	38,025 (14.5%)	3146 (9.4%)	36,870 (15.9%)
Geography				
Urbanicity ^l^				
Large metropolitan area	4478 (33.3%)	44,989 (24.8%)	9136 (39.6%)	36,905 (22.5%)
Small metropolitan area	7778 (43.4%)	56,013 (29.1%)	8009 (33.0%)	51,083 (29.5%)
Micropolitan area	2268 (14.2%)	47,101 (22.3%)	4040 (16.0%)	42,848 (22.9%)
Rural area	1966 (9.1%)	52,622 (23.8%)	3121 (11.4%)	49,477 (25.1%)
Type of community care received ^m^				
Medicine subspecialty	4521 (18.7%)	61,841 (23.3%)	6670 (20.0%)	57,071 (24.0%)
Surgical care	3473 (20.2%)	44,929 (21.9%)	5987 (23.0%)	40,205 (21.8%)
Eye care	2344 (11.9%)	25,997 (13.4%)	2893 (12.3%)	24,068 (13.8%)
Acupuncture	3495 (32.5%)	37,267 (27.9%)	5872 (33.0%)	31,409 (26.7%)
Psychiatric	747 (9.2%)	5199 (4.9%)	781 (5.8%)	4431 (4.8%)
Primary care	727 (3.4%)	8411 (3.4%)	567 (1.9%)	8054 (3.7%)
Other	1183 (4.1%)	17,081 (5.2%)	1536 (4.1%)	15,075 (5.1%)

^a^Table shows characteristics of Veterans included in the Survey of Healthcare Experience of Patients (SHEP) survey, which is administered to Veterans who used VA community care in the prior 90 days. Sample characteristics are stratified by ethnicity and race. For continuous variables (e.g., age), survey-weighted means and standard deviations [brackets] are presented. For categorical variables (e.g., gender), unweighted frequencies and survey-weighted proportions (parentheses) are presented. Weighted means and proportions calculated using SHEP survey weights. Characteristics are for pooled data from 2016 to 2021

^b^Analyses of Veterans categorized by ethnicity included all racial groups. Analyses of Veterans categorized as Black, African American, or White included all ethnic groups. Race and ethnicity were self-reported by respondents to the SHEP survey

^c^Weighted sample size calculated using SHEP survey weights

^d^Self-reported by SHEP respondents

^e^Count of 0–30 comorbidity indicators in the Elixhauser Comorbidity Index. Comorbidities assessed from diagnoses in VA corporate data warehouse (for care delivered in VA facilities) or VA Program Integrity Tool File (for VA-funded community care) during the two federal fiscal years preceding the SHEP survey. All Veterans for whom data on comorbid conditions were utilized were present in the VA system for at least 2 years prior to the survey

^f^Diagnosis of substance use disorder related to drug or alcohol use, assessed from diagnoses in VA corporate data warehouse and VA Program Integrity Tool File for Veterans during the two federal fiscal years preceding the SHEP survey

^g^Diagnosis of bipolar disorder, major depression, post-traumatic stress disorder, schizophrenia, or psychosis, assessed from diagnoses reported in VA corporate data warehouse and VA Program Integrity Tool File captured for Veterans during the two federal fiscal years preceding the SHEP survey

^h^Data from the VA CDW. Veterans were categorized by the VA into 1 of 8 priority groups based on military service, disability, income, and other benefit factors. Priority groups 1–4 include Veterans with the most significant levels of service-connected disability. Priority group 5 includes Veterans with low incomes without service-connected disabilities. Priority group 6 includes Veterans seeking care for radiation, toxic substances, or other environmental exposures. Priority groups 7 and 8 include Veterans with non-service-connected disabilities who are required to make copayments for VA care

^i^Data from Medicaid enrollment files linked to VA administrative data and include Medicaid enrollment in the two federal fiscal years preceding the SHEP

^j^Data from Medicare enrollment files linked to VA administrative data and include Medicare Parts A and B (both fee-for-service Medicare and Medicare Advantage Plans) enrollment data from the two federal fiscal years preceding the SHEP

^l^Urbanicity assessed from Department of Agriculture Rural–Urban Commuting Area (RUCA) codes, which were linked to Veterans by residential ZIP code

^m^VA SHEP respondents were stratified by the type of community care received during the prior 90 days. We used detailed information on community care services to group Veterans into 7 categories that differentiate between types of community care received

### Unadjusted Analyses

**Figure 1:**
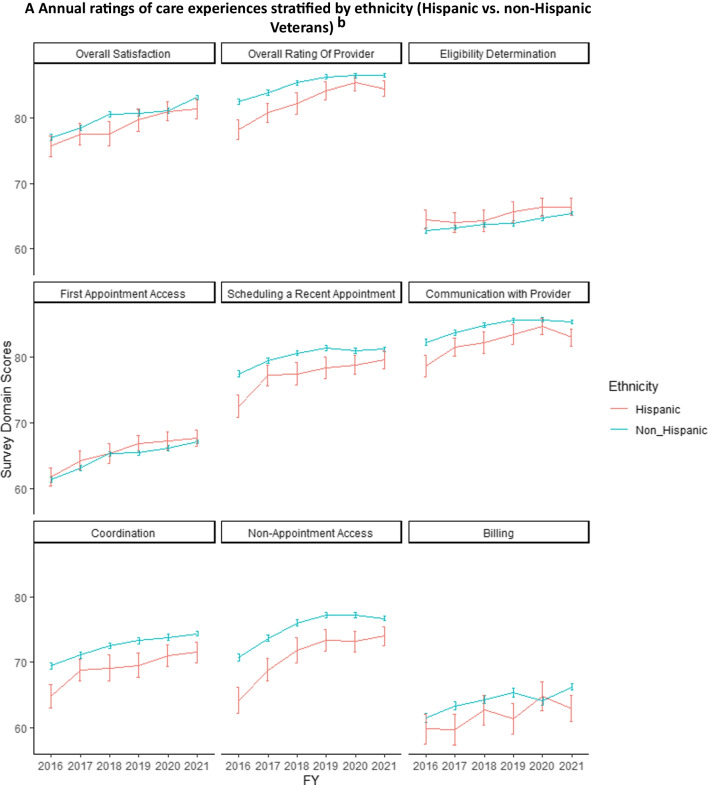

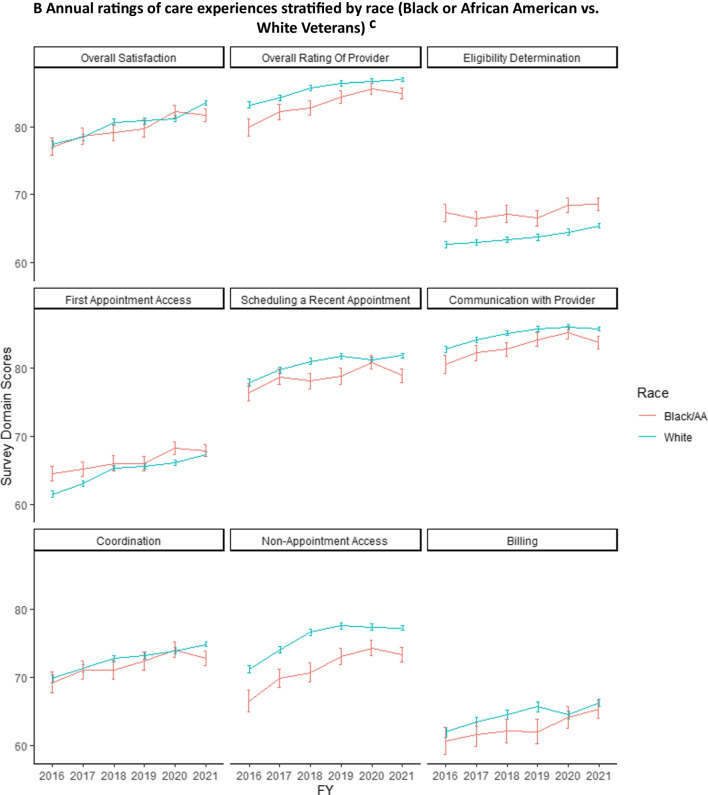
Unadjusted annual ratings of Veterans’ experiences with VA community care, stratified by ethnicity and race, 2016–2021^a^*.* **A** Annual ratings of care experiences stratified by ethnicity (Hispanic vs. non-Hispanic Veterans)^b^*.* **B** Annual ratings of care experiences stratified by race (Black or African American vs. White Veterans)^c^. ^a^Graphs display annual unadjusted mean ratings of Veterans’ experiences with VA community care by survey domain, assessed from the VA Survey of Healthcare Experience of Patients (SHEP) survey. Mean ratings are stratified by ethnicity (**A**) and race (**B**). Estimates are weighted using survey weights. 95% confidence bars were calculated using heteroskedasticity-robust standard errors. All scores are linearly transformed onto a common 100-point scale See “METHODS” and Appendix for definitions of survey domains and calculation of scores. ^b^Analyses of Veterans categorized by ethnicity included all racial groups. Ethnicity was self-reported by SHEP survey respondents. ^c^Analyses of Veterans categorized as Black, African American, or White included all ethnic groups. Race was self-reported by SHEP survey respondents.

In unadjusted analyses of community care ratings from 2016 to 2021, Hispanic Veterans had lower ratings of care than non-Hispanic Veterans in overall provider ratings, scheduling a recent appointment, provider communication, care coordination, and non-appointment access (Fig. 1A), and billing. Ratings for eligibility determination, first appointment access, and billing were lower overall and did not differ significantly between Hispanic and non-Hispanic Veterans (indicated by overlapping confidence intervals in multiple years). Lower ratings were also observed for Black than White Veterans in overall provider ratings, provider communication, and non-appointment access (Fig. 1B). Black Veterans reported higher ratings navigating eligibility determinations than White Veterans, although ratings in this domain were low for both groups. Both Black and White Veterans reported relatively low and similar ratings for first appointment access and billing. Across domains, mean ratings improved by 2.4 to 6.4 points (on a 100-point scale) from 2016 to 2021. Racial and ethnic differences in ratings of care persisted over time between non-Hispanic and White Veterans vs. those from underrepresented ethnic and racial groups.

### Adjusted Analyses

In Model 1, Hispanic Veterans had significantly lower ratings of their health care experiences in the following domains: overall rating of community care provider, scheduling a recent appointment, provider communication, care coordination, non-appointment access, and billing (Table 2). Black Veterans had significantly lower ratings of care than White Veterans in overall ratings of community care providers, scheduling a recent appointment, provider communication, non-appointment access, and billing (Table 3). For example, overall ratings of community care providers were lower among Hispanic Veterans than non-Hispanic Veterans (difference, − 1.36 points; 95% CI − 1.97 to − 0.75) and among Black Veterans than White Veterans (difference, − 1.71 points; 95% CI − 2.16 to − 1.25). Conversely, Hispanic and Black Veterans had higher ratings of their experiences with eligibility determination and scheduling an initial appointment than non-Hispanic and White Veterans.

**Table 2:** Adjusted Differences in Veterans’ Experiences with VA Community Care Between Hispanic and Non-Hispanic Veterans, 2016–2021 ^a^

		Adjusted differences between Hispanic vs. non-Hispanic Veterans^a^					
		Model 1: Adjusted for demographics, health status, and type of community care received		Model 2: Adjusted for Model 1 covariates plus geography		Model 3: Adjusted for Model 2 covariates plus insurance and SES	
Domain	Mean (SD) score^b^	Adjusted difference (95% CI)^d^	*p*-value	Adjusted difference (95% CI)^c^	*p*-value	Adjusted difference (95% CI)^c^	*p*-value
Overall satisfaction with VA community care	81.08 (25.18)	− 0.21 (− 0.95, 0.53)	0.58	− 0.28 (− 1.08, 0.52)	0.50	− 0.25 (− 1.05, 0.56)	0.55
Overall rating of community care provider	85.63 (20.69)	− 1.36 (− 1.97, − 0.75)	< 0.001	− 0.80 (− 1.46, − 0.14)	0.02	− 0.77 (− 1.44, − 0.11)	0.02
Satisfaction with eligibility determination process for VA community care	64.74 (23.92)	2.83 (2.15, 3.50)	< 0.001	2.56 (1.84, 3.29)	< 0.001	2.48 (1.76, 3.21)	< 0.001
Satisfaction with getting first VA community care appointment	66.14 (21.87)	2.01 (1.39, 2.63)	< 0.001	2.04 (1.37, 2.71)	< 0.001	2.01 (1.34, 2.67)	< 0.001
Satisfaction with scheduling recent appointment for VA community care	80.67 (24.11)	− 1.72 (− 2.41, − 1.03)	< 0.001	− 1.32 (− 2.06, − 0.58)	0.001	− 1.28 (− 2.02, − 0.54)	0.001
Rating of VA community care provider’s communication	85.05 (21.78)	− 1.67 (− 2.30, − 1.03)	< 0.001	− 0.99 (− 1.67, − 0.31)	0.004	− 0.94 (− 1.62, − 0.26)	0.01
Rating of care coordination	73.08 (27.03)	− 1.25 (− 2.03, − 0.47)	0.002	− 0.15 (− 0.99, 0.69)	0.73	− 0.14 (− 0.97, 0.70)	0.75
Satisfaction with timely access (other than scheduling an appointment)	76.06 (26.22)	− 3.80 (− 4.55, − 3.06)	< 0.001	− 2.42 (− 3.21, − 1.63)	< 0.001	− 2.30 (− 3.10, − 1.51)	< 0.001
Satisfaction with billing and out-of-pocket payments for VA community care	64.55 (37.19)	− 2.40 (− 3.43, − 1.37)	< 0.001	− 2.65 (− 3.75, − 1.55)	< 0.001	− 2.59 (− 3.70, − 1.49)	< 0.001

^a^Analyses of Veterans categorized by ethnicity included all racial groups. Ethnicity was self-reported by SHEP survey respondents

^b^Mean and standard deviations of scores for each domain of community care experiences. Estimates constructed using SHEP survey weights and estimated among all Veterans in our study for the period 2016–2021

^c^Adjusted differences represent the difference between (1) Hispanic vs. non-Hispanic Veterans (all racial groups) and (2) Black or African vs. White Veterans (all ethnic groups), pooled across study years. Estimates from a respondent-level linear regression model that predicted each domain score as a function of an indicator of Hispanic ethnicity or Black race, adjusting for the covariates as indicated in the table column and year fixed effects. See “METHODS” and Table 1 for descriptions of these covariates. Adjusted differences are linear differences on a 100-point scale. Dividing the adjusted difference by the standard deviation of the score gives the difference relative to the distribution of respondent-level domain scores (i.e., an effect size). Estimates are weighted using SHEP survey weights. 95% confidence intervals and *p*-values calculated using heteroskedasticity-robust standard errors

**Table 3:** Adjusted Differences in Veterans’ Experiences with VA Community Care Between Black/African American and White Veterans, 2016–2021

		Adjusted differences between Black or African American vs. White Veterans^a^					
		Model 1: Adjusted for demographics, health status, and type of community care received		Model 2: Adjusted for Model 1 covariates plus geography		Model 3: Adjusted for Model 2 covariates plus insurance and SES	
Domain	Mean (SD) score^b^	Adjusted difference (95% CI)^d^	*p*-value	Adjusted difference (95% CI)^c^	*p*-value	Adjusted difference (95% CI)^c^	*p*-value
Overall satisfaction with VA community care	81.08 (25.18)	− 0.08 (− 0.63, 0.47)	0.77	0.60 (0.00, 1.19)	0.05	0.61 (0.02, 1.21)	0.04
Overall rating of community care provider	85.63 (20.69)	− 1.71 (− 2.16, − 1.25)	< 0.001	− 1.33 (− 1.82, − 0.84)	< 0.001	− 1.28 (− 1.77, − 0.79)	< 0.001
Satisfaction with eligibility determination process for VA community care	64.74 (23.92)	4.79 (4.26, 5.33)	< 0.001	5.12 (4.55, 5.69)	< 0.001	5.11 (4.54, 5.68)	< 0.001
Satisfaction with getting first VA community care appointment	66.14 (21.87)	1.99 (1.50, 2.48)	< 0.001	3.18 (2.66, 3.70)	< 0.001	3.22 (2.70, 3.74)	< 0.001
Satisfaction with scheduling recent appointment for VA community care	80.67 (24.11)	− 1.99 (− 2.53, − 1.45)	< 0.001	− 1.33 (− 1.90, − 0.75)	< 0.001	− 1.21 (− 1.78, − 0.63)	< 0.001
Rating of VA community care provider’s communication	85.05 (21.78)	− 1.61 (− 2.09, − 1.13)	< 0.001	− 1.21 (− 1.72, − 0.69)	< 0.001	− 1.14 (− 1.66, − 0.62)	< 0.001
Rating of care coordination	73.08 (27.03)	0.36 (− 0.25, 0.96)	0.25	1.26 (0.61, 1.91)	< 0.001	1.41 (0.75, 2.06)	< 0.001
Satisfaction with timely access (other than scheduling an appointment)	76.06 (26.22)	− 4.49 (− 5.07, − 3.90)	< 0.001	− 3.41 (− 4.03, − 2.79)	< 0.001	− 3.36 (− 3.99, − 2.74)	< 0.001
Satisfaction with billing and out-of-pocket payments for VA community care	64.55 (37.19)	− 2.20 (− 3.01, − 1.40)	< 0.001	− 2.16 (− 3.01, − 1.30)	< 0.001	− 2.48 (− 3.33, − 1.62)	< 0.001

^a^Analyses of Veterans categorized as Black, African American, or White included all ethnic groups. Race was self-reported by SHEP survey respondents

^b^Mean and standard deviations of scores for each domain of community care experiences. Estimates were constructed using SHEP survey weights and estimated among all Veterans in our study for the period 2016–2021

^c^Adjusted differences represent the difference between (1) Hispanic vs. non-Hispanic Veterans (all racial groups) and (2) Black or African vs. White Veterans (all ethnic groups), pooled across study years. Estimates from a respondent-level linear regression model that predicted each domain score as a function of an indicator of Hispanic ethnicity or Black race, adjusting for the covariates as indicated in the table column and year fixed effects. See “METHODS” and Table 1 for descriptions of these covariates. Adjusted differences are linear differences on a 100-point scale. Dividing the adjusted difference by the standard deviation of the score gives the difference relative to the distribution of respondent-level domain scores (i.e., an effect size). Estimates are weighted using SHEP survey weights. 95% confidence intervals and *p*-values calculated using heteroskedasticity-robust standard errors

For most domains of community care experiences, estimated differences in community care ratings by race and ethnicity did not differ appreciably between Models 1, 2, and 3. In Models 2 and 3, both Hispanic and Black Veterans had lower ratings of care than non-Hispanic and White Veterans, respectively, in overall rating of community providers, scheduling a recent appointment, provider communication, non-appointment access, and billing. For example, ratings of non-appointment access were lower among Black than White Veterans (difference in Model 3, − 3.35 points; 95% CI − 3.97 to − 2.73) and Hispanic Veterans than non-Hispanic Veterans (difference in Model 3, − 2.37 points; 95% CI − 3.17 to − 1.58). Expressed relative to the SD of this domain score (26.22 points), these differences correspond to an effect size of − 0.13 SDs among Black Veterans and − 0.09 SDs among Hispanic Veterans.

Similar to Model 1, in Models 2 and 3, Hispanic and Black Veterans had higher ratings than non-Hispanic and White Veterans, respectively, of eligibility determination and scheduling a first appointment. However, in Models 2 and 3 (compared to Model 1), we no longer found a significant difference in care coordination ratings between Hispanic and non-Hispanic Veterans, whereas we found a slightly higher adjusted care coordination rating among Black Veterans than White Veterans (difference in Model 3, 1.4 points; 95% CI 0.75 to 2.06). From Model 1 to Model 3, differences in care coordination ratings between Hispanic vs. non-Hispanic Veterans narrowed from − 1.25 to − 0.14 points, and widened from 0.36 to 1.41 points between Black vs. White Veterans. Our results did not differ appreciably in a sensitivity analysis that controlled for individual Elixhauser comorbidities (Appendix Tables 6 and 7).

### Adjusted Analyses of High vs. Low Ratings of Community Care Experiences

**Figure 2:**
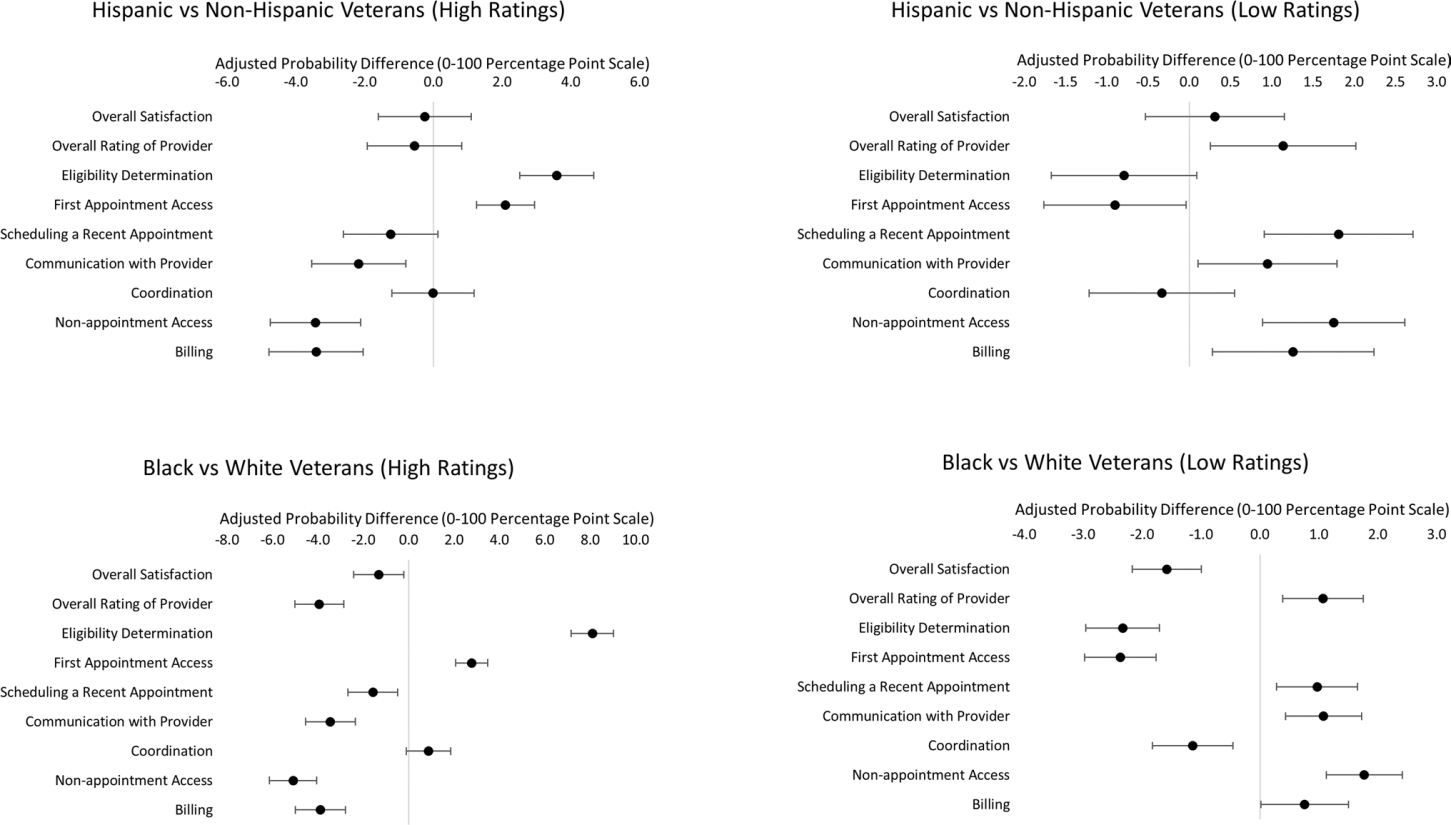
Forest plots of adjusted marginal differences in the probability of reporting high and low ratings of care between Hispanic vs. non-Hispanic Veterans and Black vs. White Veterans with VA Community Care, 2016–2021^a^. ^a^Each panel in this plot displays the adjusted marginal differences in the probability that Veterans who identify as Hispanic or Black report positive or negative community care experiences, relative to Veterans who identify as non-Hispanic or White (respectively)). Estimates use pooled data from 2016 to 2021. Estimates are obtained from a respondent-level logistic regression model that predicted each domain probability difference for positive or negative community care experiences as a function of Hispanic ethnicity or Black race, adjusting for all covariates indicated in Table 1 and year fixed effects. From these logistic regression models, we estimated the marginal difference (on the 0–100 percentage point scale) in the probability of the outcome. Positive experiences of care were defined by high ratings equivalent to or higher than the 90th percentile of the domain score distribution (among all SHEP survey respondents in our sample) and study years. Negative experiences of care are defined by low ratings equivalent to or lower than the 10th percentile of the distribution of the domain score (among all SHEP survey respondents in our sample) and study years. Because the distribution of some scores is discrete, a rating equivalent to or higher than the 90th percentile or lower than the 10th percentile may include more than 10% of Veterans.

In logistic regression models examining the binary outcome of high vs. low ratings of community care, Hispanic Veterans were less likely than non-Hispanic Veterans to report high ratings of provider communication, non-appointment access, and billing (Fig. 2). Similarly, Hispanic Veterans were more likely than non-Hispanic Veterans to report low ratings of their provider overall, scheduling a recent appointment, provider communication, non-appointment access, and billing. Conversely, Hispanic Veterans were more likely than non-Hispanic Veterans to report high ratings of navigating community care eligibility determination and scheduling initial appointments. Black Veterans were more likely than White Veterans to report low ratings with overall provider experience, scheduling a recent appointment, provider communication, non-appointment access, and billing, and were more likely to report high ratings for eligibility determination and scheduling an initial community care appointment.

## DISCUSSION

This study examined Veterans’ self-reported experiences with VA-funded community care from 2016 to 2021 using national data from respondents to the VA SHEP-CC survey. We had three principal findings. First, Black and Hispanic Veterans reported lower ratings of care in five domains compared to White and non-Hispanic Veterans. Specifically, Black and Hispanic Veterans reported lower ratings than their White and non-Hispanic Veteran counterparts in overall provider ratings, scheduling a recent appointment, provider communication, non-appointment access, and billing. These disparities were statistically significant, although they were quantitatively small (equivalent to − 0.04 to − 0.12 standard deviations of domain scores). Second, Black and Hispanic Veterans both reported better ratings than White and non-Hispanic Veterans in eligibility determination and first appointment access. However, Veterans from all racial and ethnic groups rated their care less favorably in these domains compared to other domains. In fully adjusted models, Black Veterans also reported better ratings of care coordination compared to White Veterans. Third, overall ratings of healthcare experiences improved over the study period for all domains. However, observed racial and ethnic disparities persisted over time.

These findings add to literature documenting racial and ethnic disparities in Veterans’ health care experiences and illuminate the extent to which disparities arise in VA community care. One study found that rural-dwelling Veterans reported worse experiences with community care vs. with care at VA facilities.^18^ Another study using SHEP data found that Veterans reported better experiences in VA facilities than in community settings in most domains, except for access to specialists.^19^ Our findings are consistent with research that identified disparities in care experiences within the VA healthcare system^7^ and among Veterans who used care outside of the VA through insurance programs such as Medicare.^35^ It is unclear whether the magnitudes of disparities within VA community care are larger or smaller than in the VA; this will be important for policymakers to monitor.

Notably, Black and Hispanic Veterans consistently rated their care worse than White Veterans for both overall provider ratings and provider communication. Prior studies of the VA healthcare system and non-VA providers also found racial and ethnic disparities in patient-reported experiences with provider communication.^7,36,37^ In studies of healthcare interactions, implicit racial bias and negative stereotypes were found to be associated with poorer communication and ratings of care, particularly among Black patients.^38^ Although the importance of policies to address systemic bias in health care is not limited to VA community care, such policies warrant particular attention in VA community care because of the unique medical and social circumstances surrounding military service.^2^ Efforts to promote culturally competent care that improves Veterans’ interactions with community-based providers could reduce disparities in these patient experience domains.

Our analyses also highlight opportunities for VA to improve certain aspects of community care eligibility and administration. To receive community care, Veterans must navigate complex program rules, requiring proof of eligibility to receive community care based on various criteria such as travel distance. Although VA has established systems to manage authorizations, referrals, and billing, Veterans have reported that these processes can be difficult to navigate.^39^ While community care experiences improved over time, Veterans across racial and ethnic groups remained less satisfied with their experiences navigating community care eligibility, scheduling appointments, and billing, compared to their ratings in other domains. Furthermore, while disparities in Veterans’ experiences with community care were often small, our analyses often revealed a consistent pattern of inequities. For example, Black and Veterans reported lower mean ratings of providers and communication than White and Veterans. Further, Black Veterans were less likely than White Veterans to report positive experiences and were more likely to report negative experiences in both of these domains. Reducing these disparities—in addition to improving overall levels of community care experience—could improve how VA community care programs serve Veterans.

### Limitations

This study had several limitations. First, because the sample was limited to Veterans who used community care, we were unable to observe factors that could have contributed to racial or ethnic differences in referrals or access to community care. Second, although we studied Veterans from different racial and ethnic groups, we were unable to explore differences in experiences by types of community care received because of sample size limitations. Third, because of small sample sizes, we were unable to examine disparities jointly by race and ethnicity (e.g., among Veterans identifying as Black and Hispanic). Further, due to the smaller representation of Veterans of American Indian, Alaska Native, or Asian backgrounds, we were unable to analyze Veterans from these less common racial and ethnic groups or those with multiracial and multiethnic backgrounds. Fourth, we could not measure provider characteristics or control for provider group effects due to the lack of provider-level data in the SHEP-CCS. Further research is needed to examine provider-level factors, such as racial and ethnic concordance, that may mediate disparities in healthcare experiences.

## CONCLUSION

Black and Hispanic Veterans reported less favorable experiences with VA-funded community care than White and non-Hispanic Veterans respectively in overall provider ratings, scheduling a recent appointment, provider communication, non-appointment access, and billing. Although quantitatively small, observed disparities persisted over time. Interventions to improve Black and Hispanic Veterans’ healthcare experience, including in areas related to patient-provider communication, could help advance equity in VA community care and the overall care of the Veteran population.

### Supplementary Information

Below is the link to the electronic supplementary material.

Supplementary file1 (DOCX 38 KB)

## Data Availability

Restricted-use data for this study were provided through a Data Use Agreement from the Veterans Health Administration.
