# Supplementary material for: Racial and Ethnic Differences in Health Care Experiences for Veterans Receiving VA Community Care from 2016 to 2021
Source: J Gen Intern Med. 2024 May 31;39(12):2249–60. doi: 10.1007/s11606-024-08818-3 (PMC11347541; doi:10.1007/s11606-024-08818-3)
Supplement: Supplementary file 1 — Supplementary file1 (DOCX 38 KB) [file 11606_2024_8818_MOESM1_ESM.docx]

**Appendix**

### Appendix Table 1: Cohort Selection Flow Table

| **Step** | **Criteria** | **# Observations Meeting Criteria** | | | |
| --- | --- | --- | --- | --- | --- |
| 1 | SHEP overall data (FY2016-2021) | 761,347 | | | |
| 2 | Include the respondents who completed the survey (comp_cc=1) | 233,634 | | | |
| 3 | Observations are in CDW ( "RemoveRecord=0" ) | 233,446 | | | |
| 4 | Exclude US territories* (Primary StaPa) | 232,353 | | | |
| 5 | Drop missing values of physicians_per_1000, HPSA and RUCA | **231,869** | | | |
|  |  | **Comparison by Ethnicity** | | **Comparison by Race** | |
|  |  | **Hispanic** | 16,490 | **Black/African American** | 24,306 |
|  |  | **Non-Hispanic** | 200,725 | **White** | 180,313 |
|  |  | **Excluded** | 14,654 | **Excluded** | 27,250 |

* US territories include the Virgin Islands, America Samoa, Puerto Rico, Guam, and the Northern Mariana Islands.

### Appendix Table 2: Definitions of domain scores and their construction

| **MEASURE** | **RESPONSE SCALE^a^** | **QUESTION(S)** |
| --- | --- | --- |
| Overall Satisfaction | Very dissatisfied, Dissatisfied, Somewhat dissatisfied, Somewhat satisfied, Satisfied, Very satisfied | Q40. Overall, how satisfied are you with your VA Community Care? |
| Overall Rating of Provider | 0, 1, 2, 3, 4, 5, 6, 7, 8,  9, 10 | Q35. Using any number from 0 to 10, where 0 is the worst provider possible and 10 is the best provider possible, what number would you use to rate your VA Community Care provider? |
| Eligibility Determination | Strongly disagree, Disagree, Neither agree nor disagree, Agree, Strongly agree | Q3. The eligibility requirements for VA Community Care are clear. |
|  |  | Q4. The information available about eligibility for VA Community Care is helpful. |
| First Appointment Access | Strongly disagree, Disagree, Neither agree nor disagree, Agree, Strongly agree | Q5. The process for scheduling my first appointment for this service was clearly explained to me. |
|  |  | Q6. It was clear who was responsible for the process of arranging my first appointment for this service. |
|  |  | Q7. I had enough say in selecting a VA Community Care provider for this service. |
|  |  | Q8. I had enough say in selecting the date and time of my first appointment for this service. |
|  |  | Q9. I was able to get my first appointment for this service as soon as I needed. |
|  |  | Q10. It was easy to get my first appointment for this service. |
|  |  | Q11. I understand the process for getting VA Community Care, including determining eligibility, finding a community provider, and scheduling an appointment. |
| Scheduling a Recent Appointment | Never, Sometimes, Usually, Always | Q13. In the last 3 months, how often did you get an appointment for this service as soon as you needed? |
|  |  | Q14. In the last 3 months, how often were you able to get an appointment for this service at a convenient date and time? |
|  |  | Q15. In the last 3 months, how often were you able to receive this service at a convenient location? |
| Communication with Provider | Never, Sometimes, Usually, Always | Q17. In the last 3 months, how often did your VA Community Care provider explain things in a way that was easy to understand? |
|  |  | Q18. In the last 3 months, how often did your VA Community Care provider listen carefully to you? |
|  |  | Q20. In the last 3 months, how often did your VA Community Care provider give you easy to understand information about these health questions or concerns? |
|  |  | Q25. In the last 3 months, how often did your VA Community Care provider show respect for what you had to say? |
|  |  | Q26. In the last 3 months, how often did your VA Community Care provider spend enough time with you? |

^a^ All items were converted to a 0-100 scale prior to aggregation to domain scores. For example, for an item that used a 6-level scale (1-6), we used the following formula to convert scores: [(response level)-1/(6-1)]*100

### Appendix Table 3: Unadjusted distributions of domain scores pooled across racial/ethnic groups

|  | Mean | Std. dev. |
| --- | --- | --- |
| Overall_Satisfaction | 81.0793 | 25.1809 |
| OverallRating_Of_Provider | 85.6313 | 20.6890 |
| Eligibility_Determination | 64.7437 | 23.9157 |
| First_Appointment_Access | 66.1359 | 21.8679 |
| Scheduling a Recent Appointment | 80.6672 | 24.1132 |
| Communication_with_Provider | 85.0534 | 21.7838 |
| Coordination | 73.0805 | 27.0262 |
| Non_Appointment_Access | 76.0612 | 26.2220 |
| Billing | 64.5501 | 37.1858 |

### Appendix Table 4: Frequency of Survey Completion

| **Multi_Survey** | **Freq.** | **Percent** |
| --- | --- | --- |
| Once | 175,460 | 75.67 |
| Multiple | 56,409 | 24.33 |
| Total | 231,869 | 100 |

### Appendix Table 5 Unadjusted mean (standard deviation) of care in each domain, and the Black-White and Hispanic-Non-Hispanic difference (standard deviation) in 2016 and 2021

|  | **Black** | **White** | **Black-White Diff** | **Hispanic** | **Non-Hispanic** | **Hispanic-Non-Hispanic Diff** |
| --- | --- | --- | --- | --- | --- | --- |
| **Overall Satisfaction** |  |  |  |  |  |  |
| 2016 | 77.08(66.58) | 77.64(65.31) | -0.38(0.72) | 75.88(61.5) | 77.17(65.57) | -1.37(0.82) |
| 2021 | 81.77(19.42) | 83.63(19.3) | -1.82(0.52) | 81.27(20.13) | 83.27(19.21) | -1.81(0.79) |
| **Overall Rating of Provider** |  |  |  |  |  |  |
| 2016 | 79.78(60.3) | 83.25(55.03) | -3.29(0.68) | 78.37(58.86) | 82.56(55.72) | -4.28(0.79) |
| 2021 | 84.89(16.63) | 86.97(15.92) | -2.03(0.45) | 84.42(17.22) | 86.55(16.04) | -2.04(0.64) |
| **Eligibility Determination** |  |  |  |  |  |  |
| 2016 | 67.48(62.38) | 63.094(58.4) | 4.66(0.69) | 64.94(59.03) | 63.13(58.8) | 1.71(0.78) |
| 2021 | 68.83(20.1) | 65.61(18.78) | 3.17(0.51) | 66.54(19.54) | 65.66(18.92) | 0.99(0.69) |
| **First Appointment Access** |  |  |  |  |  |  |
| 2016 | 64.77(56.73) | 62.043(55.16) | 3.03(0.61) | 62.33(55.26) | 61.95(55.28) | 0.37(0.74) |
| 2021 | 68.38(18.25) | 67.67(17.07) | 0.55(0.47) | 67.81(17.85) | 67.4(17.19) | 0.55(0.64) |
| **Scheduling A Recent Appointment** |  |  |  |  |  |  |
| 2016 | 76.44(67.56) | 78.04(63.69) | -1.44(0.71) | 72.35(67.29) | 77.64(63.83) | -5(0.91) |
| 2021 | 78.89(20.11) | 82.00(18.48) | -2.98(0.55) | 79.68(19.04) | 81.35(18.72) | -1.63(0.7) |
| **Communication with Provider** |  |  |  |  |  |  |
| 2016 | 80.62(63.19) | 82.98(56.3) | -2.27(0.73) | 78.71(61.7) | 82.53(56.75) | -3.67(0.86) |
| 2021 | 83.82(18.16) | 85.96(16.92) | -2.04(0.48) | 83.17(18.01) | 85.55(17.14) | -2.36(0.66) |
| **Coordination** |  |  |  |  |  |  |
| 2016 | 69.26(72.74) | 69.99(68.77) | -0.6(0.83) | 65.03(70.12) | 69.56(68.54) | -4.63(0.95) |
| 2021 | 72.83(21.63) | 75.03(21.06) | -2.12(0.58) | 71.53(21.52) | 74.5(21.13) | -2.83(0.82) |
| **Non-Appointment Access** |  |  |  |  |  |  |
| 2016 | 66.69(77.26) | 71.40(71.21) | -4.65(0.87) | 64.25(74.8) | 70.97(71.1) | -6.62(1.06) |
| 2021 | 73.46(21.77) | 77.51(20.24) | -3.88(0.57) | 74.22(20.96) | 76.87(20.46) | -2.71(0.78) |
| **Billing** |  |  |  |  |  |  |
| 2016 | 60.59(95.04) | 62.18(92.35) | -1.27(1.08) | 59.76(88.53) | 61.66(91.72) | -1.66(1.2) |
| 2021 | 65.5(28.36) | 66.45(29.51) | -0.92(0.73) | 62.84(28.7) | 66.36(29.15) | -3.29(1.03) |

### Appendix Table 6 Adjusted differences in Veterans’ experiences with VA community care between Hispanic and non-Hispanic Veterans, 2016-2021^a^

**Adjusted differences between Hispanic vs. non-Hispanic Veterans ^a^**

|  |  | **Model 4:** Adjusted for model 3 covariates plus individual comorbidities and survey modality | |
| --- | --- | --- | --- |
| Domain | Mean (SD) score ^c^ | Adjusted difference (95% CI) ^d^ | P-value |
| Overall satisfaction with VA community care | 81.08 (25.18) | -0.29 (-1.09, 0.51) | 0.4803 |
| Overall rating of community care provider | 85.63 (20.69) | -0.79 (-1.45, -0.13) | 0.0193 |
| Satisfaction with eligibility determination process for VA community care | 64.74 (23.92) | 2.41 (1.68, 3.13) | <0.001 |
| Satisfaction with getting first VA community care appointment | 66.14 (21.87) | 1.96 (1.29, 2.63) | <0.001 |
| Satisfaction with scheduling recent appointment for VA community care | 80.67 (24.11) | -1.31 (-2.06, -0.57) | 0.0005 |
| Rating of VA community care provider’s communication | 85.05 (21.78) | -0.96 (-1.64, -0.28) | 0.0058 |
| Rating of care coordination | 73.08 (27.03) | -0.19 (-1.03, 0.65) | 0.6556 |
| Satisfaction with timely access (other than scheduling an appointment) | 76.06 (26.22) | -2.33 (-3.12, -1.53) | <0.001 |
| Satisfaction with billing and out-of-pocket payments for VA community care | 64.55 (37.19) | -2.64 (-3.75, -1.54) | <0.001 |

^a^ Analyses of Veterans categorized by ethnicity included all racial groups. Ethnicity was self-reported by SHEP survey respondents.

^b^ Analyses of Veterans categorized as Black, African American, or White included all ethnic groups. Race was self-reported by SHEP survey respondents.

^c^ Mean and standard deviations of scores for each domain of community care experiences. Estimates constructed using SHEP survey weights and estimated among all Veterans in our study for the period 2016-2021.

^d^ Adjusted differences represent the difference between 1) Hispanic vs. non-Hispanic Veterans (all racial groups) or 2) Black or African vs. White Veterans (all ethnic groups), pooled across study years. Estimates from a respondent-level linear regression model that predicted each domain score as a function of an indicator of Hispanic ethnicity or Black race, adjusting for the covariates as indicated in the table column and year fixed effects. See the methods and Table 1 for descriptions of these covariates. Adjusted differences are linear differences on a 100-point scale. Dividing the adjusted difference by the standard deviation of the score gives the difference relative to the distribution of respondent-level domain scores (i.e., an effect size). This model adjusts for individual Elixhauser comorbidity indicators instead of a linear comorbidity index. Estimates are weighted using SHEP survey weights. 95% confidence intervals and P-values calculated using heteroskedasticity-robust standard errors.

### Appendix Table 7 Adjusted differences in Veterans’ experiences with VA community care between Black/African American and White Veterans, 2016-2021^a^

**Adjusted differences between Black/African American vs. White Veterans ^a^**

|  |  | **Model 4:** Adjusted for model 3 covariates plus individual comorbidities and survey modality | |
| --- | --- | --- | --- |
| Domain | Mean (SD) score ^c^ | Adjusted difference (95% CI) ^d^ | P-value |
| Overall satisfaction with VA community care | 81.08 (25.18) | 0.43 (-0.17, 1.03) | 0.159 |
| Overall rating of community care provider | 85.63 (20.69) | -1.37 (-1.86, -0.87) | <0.001 |
| Satisfaction with eligibility determination process for VA community care | 64.74 (23.92) | 4.94 (4.36, 5.51) | <0.001 |
| Satisfaction with getting first VA community care appointment | 66.14 (21.87) | 3.05 (2.53, 3.58) | <0.001 |
| Satisfaction with scheduling recent appointment for VA community care | 80.67 (24.11) | -1.35 (-1.93, -0.77) | <0.001 |
| Rating of VA community care provider’s communication | 85.05 (21.78) | -1.22 (-1.74, -0.70) | <0.001 |
| Rating of care coordination | 73.08 (27.03) | 1.21 (0.55, 1.86) | <0.001 |
| Satisfaction with timely access (other than scheduling an appointment) | 76.06 (26.22) | -3.42 (-4.04, -2.79) | <0.001 |
| Satisfaction with billing and out-of-pocket payments for VA community care | 64.55 (37.19) | -2.43 (-3.30, -1.57) | <0.001 |

^a^ Analyses of Veterans categorized by ethnicity included all racial groups. Ethnicity was self-reported by SHEP survey respondents.

^b^ Analyses of Veterans categorized as Black, African American, or White included all ethnic groups. Race was self-reported by SHEP survey respondents.

^c^ Mean and standard deviations of scores for each domain of community care experiences. Estimates constructed using SHEP survey weights and estimated among all Veterans in our study for the period 2016-2021.

^d^ Adjusted differences represent the difference between 1) Hispanic vs. non-Hispanic Veterans (all racial groups) or 2) Black or African vs. White Veterans (all ethnic groups), pooled across study years. Estimates from a respondent-level linear regression model that predicted each domain score as a function of an indicator of Hispanic ethnicity or Black race, adjusting for the covariates as indicated in the table column and year fixed effects. This model adjusts for individual Elixhauser comorbidity indicators instead of a linear comorbidity index. See the methods and Table 1 for descriptions of these covariates. Adjusted differences are linear differences on a 100-point scale. Dividing the adjusted difference by the standard deviation of the score gives the difference relative to the distribution of respondent-level domain scores (i.e., an effect size). Estimates are weighted using SHEP survey weights. 95% confidence intervals and P-values calculated using heteroskedasticity-robust standard errors.
